# The Impact of Sleep Quality and Sleep Duration on Dietary Disorder: A Cross‐Sectional Study in Xiamen, China

**DOI:** 10.1002/fsn3.71405

**Published:** 2025-12-30

**Authors:** Hongge Tang, Kunyang Li, Qi Sun, Xiangquan Liu

**Affiliations:** ^1^ School of Public Health and Medical Technology Xiamen Medical College Xiamen Fujian People's Republic of China; ^2^ Xiamen Haicang Hospital Xiamen Fujian China; ^3^ School of Public Health China Medical University Shenyang Liaoning Province People's Republic of China

**Keywords:** adolescents, dietary disorder, sleep duration, sleep quality

## Abstract

Dietary disorder is a growing health concern among adolescents, influenced by a combination of psychosocial, behavioral, and environmental factors. This study examines the relationship between sleep quality, sleep duration, and the severity of dietary disorders in Chinese adolescents. A cross‐sectional survey was conducted among 982 adolescents in Xiamen, China, capturing data on demographics, dietary habits, and sleep health through self‐reported questionnaires. Principal component analysis (PCA) showed that dietary disorder was significantly affected by poorer subjective sleep quality and shorter sleep duration. Generalized ordered logit regression and restricted cubic splines (RCS) analyses revealed that poor sleep quality and sleep curtailment were significantly associated with higher levels of dietary disorder. Specifically, adolescents with excellent sleep quality were 58% less likely to experience dietary disorder, while those with sleep curtailment were 82% more likely to exhibit dietary disorder. The findings suggest that sleep health plays a pivotal role in shaping adolescent dietary behaviors, and interventions targeting sleep quality and duration may mitigate dietary disorders. This research provides evidence for public health initiatives focused on improving sleep hygiene as part of efforts to enhance adolescent health outcomes.

## Graphical abstract

This study investigates the relationship between sleep quality, sleep duration, and dietary disorders in adolescents in Xiamen, China. The results show that poor sleep quality and sleep curtailment are significantly associated with higher levels of dietary disorders. Sleep interventions, such as improving sleep hygiene, may help mitigate these disorders and promote healthier dietary behaviors among adolescents. Association between sleep health and dietary disorders. Excellent sleep quality and adequate duration (low PSQI score ≤ 5) are linked to a 58% lower risk, while poor sleep quality and sleep curtailment (high PSQI score > 5) are associated with an 82% higher risk of dietary disorders. Improving sleep hygiene can help reduce dietary disorder risks among Chinese adolescents.

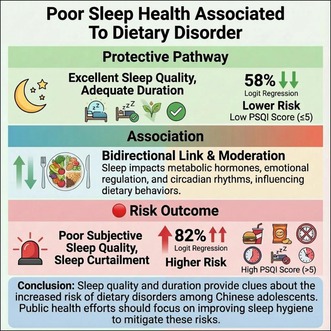

## 1 Introduction

Adolescence is a critical period characterized by rapid physical and psychological development. However, sleep health has become one of the major health concerns affecting this group. In recent years, issues related to sleep quality among adolescents have shown a progressively severe trend. A systematic review indicated that sleep curtailment or sleep disorders are prevalent among adolescents, with approximately one‐third reporting inadequate sleep duration or poor sleep quality (Cai et al. 2024). Chronic poor sleep affects adolescents' daily lives and can profoundly impact their cognitive functions, emotional regulation, and overall physical and mental health. Research has shown that sleep curtailment in adolescents is closely associated with poor school performance, depression, anxiety, and other psychological issues. Additionally, sleep disorders are linked to an increased risk of chronic diseases such as obesity and cardiovascular diseases (Wheaton et al. 2018; Talbot et al. 2010; Bacaro et al. 2020). Moreover, sleep health is usually influenced by various lifestyle factors, among which dietary patterns are considered closely related to sleep quality.

Diet plays a crucial role in daily life as food provides the essential energy, vitamins, minerals, and other nutrients needed for normal physiological functions. From birth to the end of life, obtaining adequate nutrition is necessary for growth, development, and a healthy lifespan (Wickramasinghe et al. 2020). In most modernized countries, industrial food systems have made calorie‐rich but nutrient‐poor foods widely available, leading to a food environment that favors energy toxicity (Drewnowski and Rehm 2013; Hall 2018). Currently, the mismatch between high energy and low nutritional density in food intake in Western countries is considered a major factor contributing to the rising prevalence of chronic and metabolic diseases (Poti et al. 2017). For example, in the United States, at least five of the top ten causes of death, even including COVID‐19, are diet‐related (Gropper 2023; Żarnowski et al. 2022; Centers for Disease Control and Prevention 2025). In China, the theme slogan of “healthy eating and nationwide weight loss” was introduced for 2025. Prioritizing the health of the younger population is an indispensable part of achieving the United Nations 2030 Sustainable Development Goals (Huang and Chang 2022). Since diet plays a crucial role in the development of non‐communicable diseases, improving diet quality is essential for enhancing public health.

Regular sleep and dietary behaviors are crucial for maintaining a healthy weight, improving metabolism, and enhancing cardiovascular health. However, the reality contradicts this principle. For instance, the average eating window for adolescents aged 10–18 years in the United States has exceeded 12 h (Adafer et al. 2020). A study in India found that over 30% of daily energy intake occurs in the evening and late night (Tucker et al. 2022). In the United States, from 1970 to 2009, the proportion of men and women regularly eating three meals a day decreased from 73% and 75% to 59% and 63%, respectively (Gupta et al. 2017). Some provinces and cities in China show that over 30% of children and adolescents regularly skip breakfast (Kant and Graubard 2015). These data collectively reveal a global trend of fragmented, nighttime, and less regular dietary patterns (Ren 2019). At the same time, compared to children with sufficient sleep, those who are sleep‐curtained consume 134 more kcal daily and gain about 0.22 kg in weight. No significant differences in macronutrient intake were observed, but the data suggest that changes in calorie intake may be due to an increased amount of waking time caused by sleep curtailment (Hart et al. 2013). Exploring the factors related to dietary patterns becomes increasingly important. Has sleep also become one of the influencing factors of dietary patterns, especially among adolescents?

This study is a cross‐sectional survey conducted among students from several schools in a district of Xiamen City, Fujian Province, China. It aims to explore the correlations between sleep quality, sleep duration, sleep latency, and dietary disorders among adolescents under increasing academic pressure. Xiamen presents distinct regional advantages and research significance for investigating the relationship between diet and sleep. As a typical subtropical coastal city, its warm, humid climate and residents' culturally and environmentally shaped lifestyles provide a unique context for such research. The city's diverse food culture, blending northern and southern cuisines, along with moderate urbanization and vibrant nightlife, create favorable conditions for exploring how living rhythms and sleep health influence diet. Moreover, Xiamen's many universities and diverse population allow for wide age and social representation, enhancing the study's applicability. This research aims to provide evidence for improving adolescent health and inform relevant public health policies and interventions.

## 2 Materials and Methods

### 2.1 Questionnaire Development

This study consists of three parts. The first part collects demographic information, including age, height, weight, parental education level, gender, and family residence. Sociodemographic variables often play an important role in studies on sleep and diet. The second part is a sleep questionnaire, which consists of the Pittsburgh Sleep Quality Index (PSQI), which was translated into Chinese and revised by Liu et al. in 1996 (Liu et al. 1996). It is an effective tool for measuring sleep quality and patterns. The PSQI is a self‐report questionnaire with 19 items designed to assess sleep quality over the past month. The third part includes a self‐developed questionnaire to assess adolescent dietary patterns, consisting of six items: food choices, cooking methods, late‐night snacking, social and family factors, emotions, and knowledge of healthy eating (Wei 2006; Zhang et al. 2024). A 5‐point Likert scale is used, with responses ranging from 1 (strongly disagree) to 5 (strongly agree). The total score is the sum of the six items, with higher scores indicating a greater degree of disordered eating behavior (Wei 2006).

### 2.2 Data Description

#### 2.2.1 Study Population

This cross‐sectional study was based on data collected from 1071 students across six secondary schools in Xiamen. Relevant demographic information was collected through self‐administered questionnaires. Three grade levels were randomly selected from each school, and within each grade, classes were randomly chosen using a cluster sampling method, with approximately 200 students selected per grade. Data were collected via an online survey conducted between January 5 and January 25, 2025. A total of 982 valid questionnaires were obtained, yielding a response rate of 96.56%. The study was approved by the Ethics Committee of China Medical University (approval number: [2024]059), and informed consent was obtained from both the adolescents and their parents and/or legal guardians. This study was conducted in accordance with the ethical principles of the Declaration of Helsinki, and all procedures adhered to relevant regulations and guidelines.

#### 2.2.2 Assessment of Sleep Quality, Sleep Duration and Sleep Latency

PSQI includes seven dimensions: sleep latency, sleep duration, sleep efficiency, sleep disorder, self‐rated sleep quality, the use of sleep medication, and daytime dysfunction (Boned‐Galán et al. 2022). Sleep duration is reflected by the difference between the wake‐up time in the morning and the time of falling asleep the previous night. Sleep latency is reflected by the difference between the time of getting into bed and the time of falling asleep. Higher scores indicate poorer sleep quality. A score of 0–5 indicates excellent sleep quality, 6–10 indicates satisfactory sleep quality, 11–15 indicates average sleep quality, and 16–21 indicates poor sleep quality (Chen et al. 2022). This scale has been widely used in China to assess sleep conditions and has demonstrated good reliability and validity (Liu et al. 1996).

#### 2.2.3 Assessment of Dietary Disorder

The questionnaire was designed based on a literature review and expert evaluation, with items related to dietary rhythm primarily focusing on the regularity of meals, meal timing, and the impact of emotions on dietary patterns. Through item analysis, the sensitivity, homogeneity, and representativeness of each item were assessed, and 7 unqualified items were removed. A final set of 29 items was determined, ensuring that each item effectively assessed dietary rhythm with good discriminative power and reliability.

Reliability and validity analysis was conducted by calculating Cronbach's alpha coefficient and performing confirmatory factor analysis, ensuring the questionnaire's reliability in practical applications. Subsequently, field surveys were conducted across multiple regions, with 1152 college students and 8082 middle school students completing the questionnaire. After collecting the data, further reliability and validity analysis was performed. The final Cronbach alpha coefficient of the questionnaire was 0.95, indicating very high internal consistency. The results showed that the items in the dietary rhythm dimension had high correlation and discriminative power, effectively assessing the dietary rhythm status of adolescents (Wei 2006).

This section comprises six parts, and detailed information is provided in Table S1. Each item is rated on a 5‐point Likert scale (1 = strongly disagree, 5 = strongly agree), with higher scores indicating a greater degree of disordered eating behavior. Reverse scoring is applied to items (9), (10), (11), (13), (14), and (15). The total score is obtained by summing the individual scores, with higher scores reflecting a higher degree of disordered eating behavior. Based on the total score, participants are categorized into three groups: low (below the 33.3%, < 46 points), moderate (33.3%–66.6%, 46–52 points), and high (above the 66.6%, > 52 points) levels of dietary disorder (Wei 2006; Zhang et al. 2024; Xie 2021).

#### 2.2.4 Covariates

Age, gender, physical activity, parental education level, and BMI were included as covariates.

### 2.3 Data

#### 2.3.1 Data Collection

The questionnaire survey was conducted using the online data collection platform Wenjuanxing (www.wjx.cn). To ensure data accuracy, all items were set as mandatory to guarantee questionnaire completeness, and each IP address or device was restricted to a single submission to prevent duplication. Before data collection, each student provided informed consent, and researchers were present on‐site to guide them in completing the questionnaire. The questionnaire was pre‐distributed in advance to ensure that students of this age group could fully understand its content. Subsequently, the link to the online anonymous questionnaire was sent via WeChat, a popular social media platform in China. They then forwarded the link to students through private WeChat groups. To obtain informed consent, an explanatory statement was provided at the beginning of the questionnaire. The questionnaire could be completed in approximately 20 min. The collected data from Wenjuanxing was imported into Excel for screening. A total of 1017 students clicked the link to fill out the questionnaire. After excluding responses with missing items and those with a high proportion of identical answers, a final dataset of 982 valid questionnaires was obtained and presented acceptable internal consistency (Cronbach's *α* = 0.732).

#### 2.3.2 Data Analysis

In the first step, we analyzed the relationship between sleep parameters and dietary disorder levels using multivariate statistical techniques. Seven sleep‐related variables (Subjective sleep quality, sleep duration, sleep latency, sleep efficiency, sleep disturbances, use of sleep medication, and daytime dysfunction) were included as predictors. Principal component analysis (PCA) was conducted to explore the multivariate structure of sleep‐related variables, which was performed on standardized variables, and the number of retained components was determined by eigenvalues (> 1), scree plot inspection, and cumulative explained variance. To aid interpretation, results were visualized along the first two principal components (PC1 and PC2). Data ellipses at the 95% confidence level were computed to illustrate group clustering. Loadings were normalized within each component to identify the dominant contributors.

Subsequently, restricted cubic splines (RCS), a smoothing technique, were applied to more effectively explore potential nonlinear relationships between variables. RCS was then used to visualize the overall trends between sleep quality, sleep duration and levels of dietary disorder, with the X‐axis representing sleep quality scores or sleep duration, and the Y‐axis representing dietary disorder scores. The number of knots was set to 4, which was determined based on the complexity of the data and the model fitting requirements. Typically, 4 knots is a common choice as it strikes a balance between non‐linear fitting and avoiding overfitting. Regarding the placement of the knots, the rcs() function in rms package (released by Frank E. Harrell Jr. 2021) in R automatically selects the knot locations based on the distribution of the predictor variable. In this study, the knot locations were chosen based on the quantiles of the data (e.g., 10%, 50%, and 90% quantiles) to ensure that the knots reasonably reflect the variations in the predictor variable across different intervals. The covariates included in the model were selected based on the sociodemographic factors that showed significant associations with sleep health and dietary disorder level in Tables S2 and S3. These variables (Age, gender, parental education level, and BMI) were incorporated into the model to examine their relationship with dietary disorder. We believe this method effectively captures the non‐linear relationship between sleep parameters and dietary disorder level.

Following this, we analyzed the associations between sleep quality, sleep duration, sleep latency, and dietary disorder levels. Finally, a generalized ordered logit regression model was employed to further examine the relationships between each variable and dietary disorder levels. The results were reported as odds ratios (OR) with 95% confidence intervals (CI), and we also explored the interactions between significant variables and their role in influencing dietary disorder levels, in order to provide more precise estimates of effect. To ensure the adequacy of the sample size for the generalized ordered logistic regression analysis, we conducted a power analysis using G Power (version 3.1.9.7, Heinrich Heine University Düsseldorf, German). Based on the expected effect size (Cohen's *f* ^2^ = 0.15), significance level (*α* = 0.05), and power set at 0.95 (95% probability of detecting a real effect), we calculated the required sample size to be 153. The current sample size (*n* = 982) exceeds the minimum required sample size, ensuring sufficient statistical power to detect the influence of variables on the outcome. The actual power of the analysis was found to be 0.9503, which indicates that the study has high statistical power and can reliably detect real effects.

All statistical analyses were conducted using R Statistical Software (version 4.5.1, R Core Team, New Zealand) and SPSS software (version 22.0). A two‐tailed *p* value of less than 0.05 was considered statistically significant.

## 3 Results

### 3.1 Demographic Characteristics

In this cross‐sectional study, a total of 1017 adolescents participated in the survey. After excluding questionnaires with missing responses and those with a high proportion of identical answers, 982 valid questionnaires were retained. Among the participants, 515 were male (52.44%) and 467 were female (47.56%). A total of 357 participants (36.35%) were aged 13–15 years, while 625 participants (63.65%) were aged 16–19 years. Regarding family residence, 331 participants (33.71%) lived in rural areas, while 651 (66.29%) lived in urban areas. In terms of parental education, 345 fathers (35.13%) had a junior high school degree or below, 352 (35.85%) had a high school or secondary technical school degree, 170 (17.31%) had a junior college degree, and 115 (11.71%) had a bachelor's degree or higher. Among mothers, 381 (38.8%) had a junior high school degree or below, 280 (28.51%) had a high school or secondary technical school degree, 223 (22.71%) had a junior college degree, and 98 (9.98%) had a bachelor's degree or higher. In terms of family economic status, 186 participants (18.94%) reported low financial conditions, 733 (74.64%) reported middle financial status, and 63 (6.42%) reported good financial conditions. Regarding BMI, 769 participants (78.31%) had a normal BMI (18–24), 150 (15.27%) were overweight (24–30), and 63 (6.42%) were classified as obesity (> 30), as shown in Table 1.

**TABLE 1:** Demographic characteristics.

Variables	Group	*N* (%)
Gender	Boys	515 (52.44)
	Girls	467 (47.56)
Age	13–15	357 (36.35)
	16–19	625 (63.65)
Family residence	Rural	331 (33.71)
	Urban	651 (66.29)
Paternal education level	Junior high school or below	345 (35.13)
	High school or secondary technical school	352 (35.85)
	Junior college	170 (17.31)
	Bachelor or above	115 (11.71)
Maternal education level	Junior high school or below	381 (38.8)
	High school or secondary technical school	280 (28.51)
	Junior college	223 (22.71)
	Bachelor or above	98 (9.98)
Family economic status	Low	186 (18.94)
	Middle	733 (74.64)
	Good	63 (6.42)
BMI	Normal	769 (78.31)
	Overweight	150 (15.27)
	Obesity	63 (6.42)

### 3.2 Multivariate and Principal Component Analysis of Sleep Parameters by Dietary Disorder Level

**FIGURE 1:**
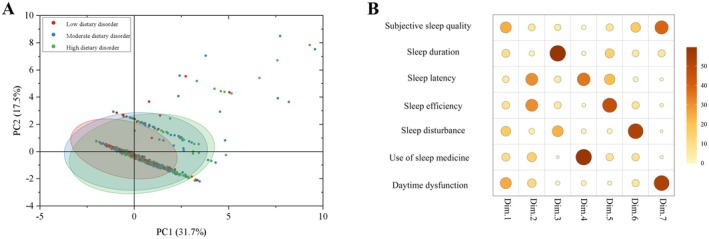
PCA‐based multivariate summary of sleep parameters across dietary disorder levels. (A) PCA score plot (PC1 vs PC2). Each point is a participant, colored by dietary‐disorder level (low, moderate, high). Axes show variance explained (PC1: 31.7%, PC2: 17.5%). Crosshairs mark the origin; shaded ellipses depict 95% data ellipses for each group. (B) Variable contribution map. Bubble heat map for Dim.1–Dim.7 where bubble size and color encode each variable's contribution to a component (loading^2^ normalized within component; %, color scale at right). Variables: Subjective sleep quality, sleep duration, sleep latency, sleep efficiency, sleep disturbance, use of sleep medicine, daytime dysfunction. “Dim.” denotes principal components (PCs).

PCA in Figure 1A revealed that participants grouped according to dietary‐disorder levels separated primarily along PC1, with partial overlap between groups. PC1 accounted for 31.7% of the variance and PC2 for 17.5%, together explaining 49.2% of the total variance. The first seven components exhibited a monotonically declining variance structure, supporting the retention of PCs up to the seventh component. Analysis of variable contributions indicated that subjective sleep quality and sleep duration were strongly represented on PC1, with moderate contributions to PC2. Higher‐order components captured additional structure: sleep efficiency contributed predominantly to PC5, sleep disturbance to PC6, and daytime dysfunction to PC7. Notably, PC4 reflected combined influences of sleep duration and use of sleep medication. Collectively, these findings suggest that PC1‐PC2 capture the primary gradient of sleep quality and quantity, while higher PCs represent more specific aspects of sleep architecture (Figure 1B).

Taken together, overall variability is driven primarily by subjective sleep quality and sleep duration, with sleep efficiency, sleep disturbance, and daytime dysfunction emerging on higher‐order components. Between‐group differences are expressed mainly along PC1, suggesting that interventions aimed at improving subjective sleep quality and ensuring adequate sleep duration are most likely to produce overall improvements in sleep status. Accordingly, we focused the subsequent analyses on sleep quality and sleep duration.

Chi‐square test results for sleep parameters and dietary disorder levels stratified by sociodemographic characteristics are provided in Tables S2 and S3.

### 3.3 Relationship Between Sleep Quality and Duration, and Dietary Disorder Levels

Results shown in Table 2 further illustrated that both sleep quality and sleep duration were significantly associated with dietary disorder levels (*p* < 0.001).

**TABLE 2:** Association between sleep health and dietary disorder level in adolescents.

Variables	Group	Dietary disorder level			*χ* ^2^ (*p*)
		Low	Moderate	High	
Sleep quality	Excellent	207	176	96	102.608 (**< 0.001** ***)
	Satisfactory	53	135	125	
	Average	15	61	58	
	Poor	11	24	21	
Sleep duration	Sleep curtailment (< 8 h)	183	282	256	35.852 (**< 0.001** ***)
	Adequate sleep (≥ 8 h)	103	114	44	
Sleep latency	< 30 min	268	365	280	0.684 (0.701)
	≥ 30 min	18	31	20	

*Note:* Bold values represents the statistical results with numeric values have been relabeled in the table.

*** *p* < 0.001.

**FIGURE 2:**
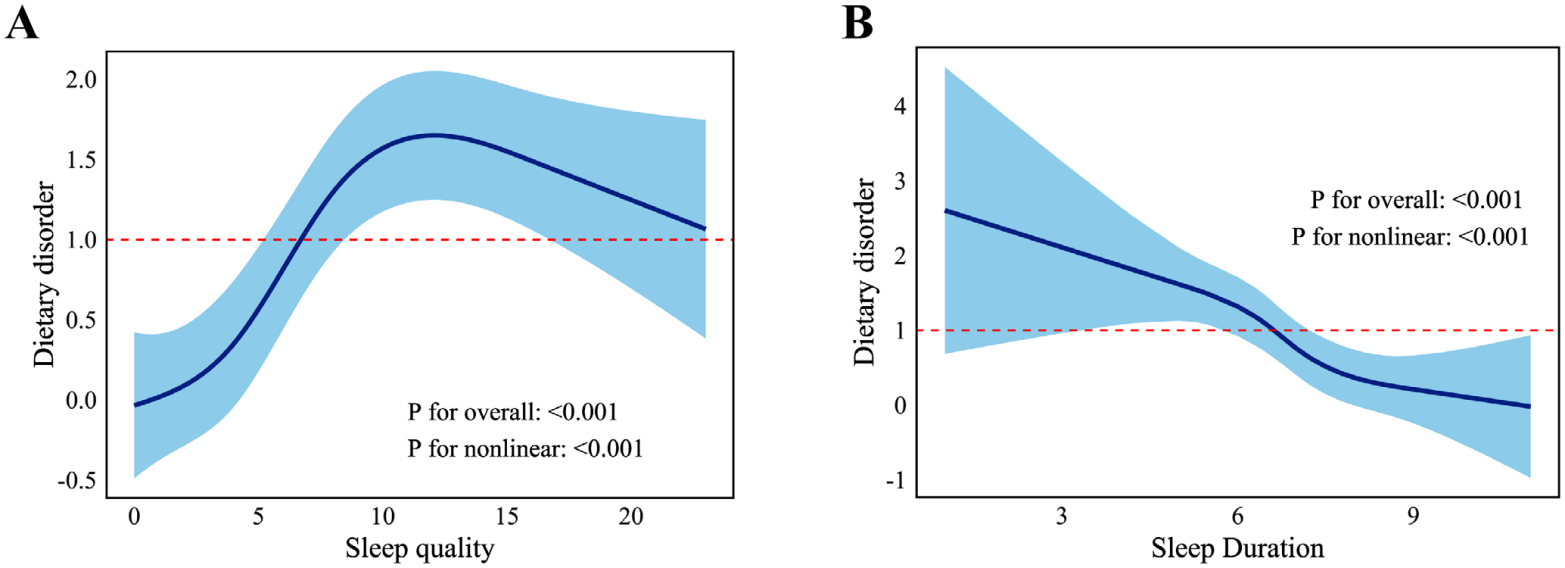
(A) The restricted cubic spline (RCS) model demonstrated a nonlinear relationship between the incidence of dietary disorder and sleep quality (*p* overall < 0.001, *p* nonlinear < 0.001). (B) The RCS model demonstrated a nonlinear relationship between the incidence of dietary disorder and sleep duration (*p* overall < 0.001, *p* nonlinear < 0.001).

The RCS curves in Figure 2 indicated a significant nonlinear association between sleep quality and dietary disorder levels after adjusting for all covariates (nonlinear *p* < 0.001, overall *p* < 0.001). As sleep quality scores increased (indicating poorer sleep quality), dietary disorder scores also increased (indicating more unhealthy eating behaviors) until reaching a specific threshold, after which the scores slightly declined, suggesting a potential saturation effect at higher sleep quality scores. Similarly, a significant nonlinear relationship was observed between sleep duration and dietary disorder levels (nonlinear *p* < 0.001, overall *p* < 0.001). Longer sleep duration was associated with lower dietary disorder levels, particularly within the range of 6–9 h, where the confidence interval was narrower.

### 3.4 Generalized Ordered Logit Regression Analysis of the Association Between Sleep Quality and Duration, and Dietary Disorder Levels

A generalized ordered logit regression was conducted using dietary disorder levels as the dependent variable (0 = low disorder group, 1 = moderate disorder group, 2 = high disorder group). Based on inclusion and exclusion criteria (*p* < 0.05), six independent variables were included in the analysis: family residence, maternal education level, daily physical exercise duration (hours), BMI, sleep quality, and sleep duration.

As shown in Table 3, the results of the generalized ordered logit regression model revealed significant associations between several variables and dietary disorder levels. Specifically, BMI was significantly associated with dietary disorder (*p* = 0.0439). Sleep quality had a significant positive impact on dietary disorder, with all levels of poorer sleep quality (excellent sleep quality, *p* = 2.44e‐13; satisfactory sleep quality, *p* = 5.60e‐11; average sleep quality, *p* = 0.000774) significantly increasing the risk of dietary disorder. In addition, sleep duration was negatively associated with dietary disorder (*p* = 0.001149), indicating that longer sleep duration has a protective effect against dietary disorder. Family residence approached significance (*p* = 0.0578), while maternal education level and gender did not have a significant impact on dietary disorder levels (*p* > 0.05). The threshold coefficients further indicated significant distinctions between dietary disorder categories, with both the 0|1 (*p* = 0.003) and 1|2 (*p* = 8.313) thresholds showing substantial effects (Table 4). These findings highlight the importance of sleep quality and sleep duration in dietary disorder prevention and suggest that BMI, although significant, has a less pronounced effect.

**TABLE 3:** Generalized ordered logit regression estimates the association between sleep health and dietary disorder.

Variables		95% LCI	OR	95% UCI	Odds ratio	*p*
Sleep duration (Reference: Adequate sleep)	Sleep curtailment	0.477	0.63	0.832	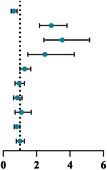	0.001**
Sleep quality (Reference: Poor)	Excellent	2.17	2.876	3.821		< 0.001 ***
	Satisfactory	2.428	3.534	5.169		< 0.001***
	Average	1.466	2.491	4.257		< 0.001***
Family residence (Reference: Urban)	Rural	0.992	1.281	1.656		0.058
Maternal education level (Reference: Bachelor or Above)	Junior high school or below	0.698	0.938	1.26		0.671
	High school or secondary technical school	0.609	0.831	1.132		0.240
	Junior college	0.707	1.087	1.674		0.705
BMI (Reference: Normal)	Overweight/Obesity	0.653	0.806	0.993		0.044*
Gender (Reference: Boys)	Girls	0.995	0.782	1.267		0.970

*Note:* Bold values represents the statistical results with numeric values have been relabeled in the table.

Abbreviations: LCI, Lower confidence interval; UCI, Upper confidence interval.

*p < 0.05; **p < 0.01; ***p < 0.001.

**TABLE 4:** Threshold estimates for dietary disorder level.

Threshold	Estimate (log‐odds)	Std. Error	*z* value
0|1	−0.49	0.16	−3.00*
1|2	1.4	0.17	8.31*

* *p* < 0.05. *z* value = log_10_ (*p*).

## 4 Discussion

This study investigated the relationships between sleep health, demographic characteristics, and dietary disorders in 982 adolescents. The key findings indicated that both sleep quality and sleep duration significantly influence adolescent dietary patterns. In addition, demographic factors such as age, family residence, maternal education level, and BMI were also significantly associated with dietary disorders.

The present findings suggest that poorer sleep characteristics may be an important risk factor for higher levels of dietary disorder. Specifically, the PCA and subsequent univariate analyses revealed that individuals with poorer subjective sleep quality, shorter sleep duration, more frequent sleep disturbances, and greater daytime dysfunction were more likely to fall into the moderate or high‐level dietary disorder levels (Irish et al. 2024; Jigeer 2024). This indicates that sleep curtailment or impaired sleep quality may affect eating behaviors and dietary control through multiple physiological and psychological pathways. On the physiological side, insufficient sleep and reduced sleep quality can disrupt the hormonal balance that regulates appetite and energy metabolism, for example, by lowering leptin levels and increasing ghrelin levels, thus intensifying cravings for high‐calorie foods (Figorilli et al. 2025; Gresser et al. 2025). Furthermore, impaired sleep may activate the hypothalamic–pituitary–adrenal (HPA) axis, elevating cortisol secretion. This not only promotes emotional eating but also disrupts insulin sensitivity, further exacerbating irregular eating patterns. On the psychological side, greater daytime dysfunction can weaken self‐control and planned eating behavior, leading individuals to opt for more convenient but nutritionally poor food choices (Ramírez‐Contreras et al. 2022).

Under these circumstances, we aim to further explore the relationship between sleep parameters and dietary disorder levels. Further analysis revealed a significant nonlinear relationship between sleep quality and dietary disorder. Declining sleep quality correlated with increased dietary disorder until a threshold, beyond which effects plateaued. This phenomenon may be related to adolescents' physiological and psychological adaptive responses. When sleep quality is inferior, the body may enter a state of stress, which in turn affects appetite and eating behavior (Griffith et al. 2024). Studies examining the association between meal timing and sleep quality have shown that individuals who experienced late‐night meals (after 9:00 p.m.) tend to have lower sleep efficiency (total sleep time/time in bed) (Iao et al. 2022). Iao and colleagues analyzed data from the American Time Use Survey involving more than 10,000 participants and also reported that those who had late‐night meals experienced more than two awakenings during the night (Gonnissen et al. 2013). Moreover, even a single night of fragmented sleep can affect appetite and may lead to prolonged food consumption. This is attributed to a reduction in morning insulin secretion and an increase in afternoon insulin secretion (*p* < 0.05), along with decreased levels of glucagon‐like peptide 1 (GLP‐1) and satiety ratings (*p* < 0.05) (Dallman 2009). However, in the present study, it was found that progressive sleep decline exacerbated disordered eating behaviors until a physiological saturation threshold was reached, beyond which the effects plateaued. This phenomenon may suggest a possible physiological and psychological “saturation” point (Ulrich‐Lai and Herman 2009), where, for example, individuals experiencing extreme fatigue might reduce their food intake or adjust their physiological needs. This could represent a biological adaptive response aimed at maintaining homeostasis (Agostini et al. 2018). However, given the exploratory nature of our study, these observations should be considered preliminary, and further research is needed to confirm whether such a “threshold effect” truly exists.

Similarly, a significant negative correlation was found between sleep duration and dietary disorder in the present study, showing that when sleep duration was between 6 and 9 h, the level of dietary disorder was relatively low. This result pointed out the importance of sleep duration in adolescent dietary health. Numerous epidemiological studies on children and adolescents have reported that shorter sleep duration or later bedtimes were associated with increased consumption of high‐energy foods (Kruger et al. 2014; Chaput et al. 2018), added sugars and sugar‐sweetened beverages (Bel et al. 2013), and decreased intake of nutrient‐rich foods such as fruits and vegetables, ultimately leading to poorer overall diet quality (Golley et al. 2013). On the other hand, excessive sleep duration may disrupt the circadian rhythm or lead to reduced physical activity due to over‐resting, which can also trigger similar health issues, including disordered dietary patterns. Previous research has revealed that adequate sleep can improve emotional eating and be more appealing to healthy food (Grandner et al. 2013; Kant and Graubard 2014; Winpenny et al. 2023). Our study also found that, in addition to sleep quality and duration, the severity of dietary disorder in adolescents was closely related to factors such as age, maternal education level, family residence, and BMI. Late adolescents (16–19 years) faced a 3.1‐fold higher dietary disorder risk versus younger peers (*p* < 0.001), reflecting pubertal neuroendocrine changes and social role transitions (Yu et al. 2025). Family education, especially maternal eating habits and health concepts, may have a significant impact on adolescents' food choices (Mora‐Urda et al. 2022; Soczewka et al. 2024). Here, our results also showed that adolescents with higher maternal education levels have lower levels of dietary disorder (*p* < 0.001). Moreover, adolescents living in rural areas exhibited 28.7% lower dietary disorder prevalence, potentially mediated by greater access to nutrient‐dense foods (e.g., seasonal produce, livestock‐derived proteins) (Heidkamp et al. 2013).

Based on the above findings, a generalized ordered logit regression analysis revealed a significant negative association between sleep quality and dietary disorder risk. Specifically, adolescents with better sleep quality demonstrated a lower risk of dietary disorder likelihood compared to those with poor sleep quality. This underscores sleep quality as a critical modulator of dietary health. Neurobiological evidence further elucidates this relationship: functional MRI studies document that sleep deprivation suppresses prefrontal cortex activity, amplifying preference for energy‐dense, nutrient‐poor foods (e.g., high‐fat snacks) while diminishing intake of fiber and lean proteins, ultimately degrading overall diet quality (Leroy and Frongillo 2007). Mechanistically, sleep disorder dysregulates appetite‐related hormones (ghrelin elevation and leptin suppression) and heightens mesocorticolimbic reactivity to food cues, synergistically driving hyperphagia (+350 kcal/day) and hedonic dietary patterns (Greer et al. 2013; Burrows et al. 2020; St‐Onge 2017). In addition, these changes in hormones and neural activities are accompanied by a greater preference for high‐calorie foods, which is mainly driven by the hedonic mechanisms of eating (Al Khatib et al. 2017; Zeron‐Rugerio et al. 2022). Consequently, poor sleep quality emerges as a modifiable obesity determinant, with meta‐analytic data indicating a 1.40‐fold elevated overweight/obesity risk among adolescents with poor sleep health (Krističević et al. 2018). Parallel analyses identified sleep duration as an independent dietary regulator. Adolescents maintaining 6–9 h/night exhibited better diet quality; notably, short sleep duration (< 6 h) disproportionately impacted children, elevating added sugar intake compared to adults (Shahdadian et al. 2023). When we further explored the interaction effects, we found that BMI significantly influenced dietary disorder, but the interaction between sleep parameters and BMI was not significant, indicating that poor sleep quality and sleep curtailment leading to dietary disorder is independent of BMI.

This study highlights a significant relationship between sleep parameters and dietary disorders, indicating that adolescents with poorer sleep quality are more likely to engage in unhealthy dietary patterns. To address this, schools could implement comprehensive sleep education programs that aim to raise awareness about the importance of maintaining good sleep hygiene. These programs could cover topics such as establishing a healthy sleep pattern, ensuring adequate sleep duration, and incorporating relaxation techniques before bedtime (Hiba et al. 2022; Michelle et al. 2021). Improving sleep quality could have a direct impact on reducing the occurrence of dietary disorders, as students who sleep better are less likely to develop unhealthy dietary patterns.

Additionally, schools can adopt a personalized approach to dietary interventions, particularly for students who experience poor sleep. For instance, providing more nutritious lunch options, limiting access to high‐sugar and high‐fat foods, and offering classroom education on the relationship between diet and sleep could significantly benefit students. Educating them about how a balanced diet can enhance sleep quality will create a positive feedback loop, where better dietary patterns lead to improved sleep, and better sleep, in turn, encourages healthier dietary patterns. Furthermore, schools could also introduce meal timing interventions, ensuring that students have a set schedule for eating, as irregular meal times have been linked to both poor sleep quality and dietary disorders. By providing targeted health education and interventions, schools can foster an environment where students not only improve their dietary patterns but also develop better sleep routines, leading to long‐term health benefits (United Nations Children's Fund 2025).

Although this study provides valuable insights into the relationship between sleep and dietary disorders, it has several limitations. First, the cross‐sectional design used in this study does not allow for the establishment of causal relationships. Future longitudinal studies would help reveal the causal links between sleep and dietary behaviors. Second, this study relied on self‐reported data, which may introduce bias. Future research could employ objective measurement tools, such as sleep monitoring devices and dietary records, to obtain more accurate data. Additionally, the PSQI tool in this study assessed sleep quality over the past month, while the focus was on current dietary habits. This temporal mismatch could affect the correlation between the two, especially since dietary habits are influenced by short‐term factors. Finally, the sample in this study was primarily from a single region of Xiamen City, so the findings cannot be extrapolated to all Chinese adolescents. Therefore, future research should expand the sample size to include adolescents from different regions and backgrounds to enhance the generalizability of the findings. Moreover, future studies could mitigate the temporal mismatch by adjusting the assessment time frame or using tools that simultaneously assess both current sleep and dietary habits.

## 5 Conclusion

This study shows clear links between sleep health (quality and duration) and adolescent dietary patterns. Improving sleep, especially among sleep‐deprived teens, may help reduce dietary disorder risks. Multivariate analysis highlights key risk factors: older age, urban living, higher BMI, and lower maternal education all increase vulnerability. These results support sleep‐focused interventions—such as circadian rhythm regulation and sleep hygiene education—as part of nutrition strategies. Efforts should target high‐risk groups, like urban and older adolescents, through school‐based sleep programs and community nutrition actions to break the sleep–diet disruption cycle.

## Author Contributions

Tang H.G.: Writing‐original draft, Investigation, Data curation, Visualization, Methodology, Formal analysis, Conceptualization; Li K.Y.: Writing‐review and editing, Investigation, Methodology; Sun Q.: Writing‐review and editing, Supervision, Conceptualization. Liu X.Q.: Funding acquisition; Investigation; Methodology; Writing‐review and editing.

## Supporting information


**Table S1:** fsn371405‐sup‐0001‐Tables.docx.

## Data Availability

The data supporting the findings of this study are available from the corresponding author upon reasonable request.
