# Supplementary material for: The Impact of Sleep Quality and Sleep Duration on Dietary Disorder: A Cross‐Sectional Study in Xiamen, China
Source: Food Sci Nutr. 2025 Dec 30;14(1):e71405. doi: 10.1002/fsn3.71405 (PMC12750510; doi:10.1002/fsn3.71405)
Supplement: Supplementary file 1 — Table S1: fsn371405‐sup‐0001‐Tables.docx. [file FSN3-14-e71405-s001.docx]

**Supplementary table 1. Structure of the dietary patterns questionnaire**

| **Section** | **Description** |
| --- | --- |
| Food choices | (1) preferences for specific foods |
|  | (2) trade-offs between taste and health when purchasing, |
|  | 1. frequency of consuming high-calorie items   (e.g., fried chicken, greasy stir-fries, fried rice) |
| Cooking methods | (4) liking for oily dishes |
|  | (5) enjoyment of dining out |
|  | (6) taste-health trade-offs when cooking |
| Late-night snacking | (7) frequency of sweet or high-calorie snacks at night |
|  | (8) having a set late-night eating schedule |
|  | (9)* frequency of skipping late-night snacks |
| Social and family factors gauge | (10)* adherence to health-related dietary restrictions despite personal preference |
|  | (11)* belief that home-cooked meals are cleaner than dining out |
| Emotional influences | (12) impact of emotions on food choices |
|  | (13)* intake and belief that emotions do not affect eating |
| Healthy eating  knowledge examines willingness | (14)* choose healthy foods despite disliking them |
|  | (15)* attention to health-related dietary information |
|  | (16) willingness to eat tasty but unhealthy foods despite knowing their risks |

Note: *, items are reverse-scored; all others are positively scored.

**Supplementary table 2. Associations between sociodemographic variables and sleep health**

| **Variables** | **Group** | **Sleep quality** | | | | **χ²** | **Sleep duration** | | **χ²** | **Sleep latency** | | **χ²** |
| --- | --- | --- | --- | --- | --- | --- | --- | --- | --- | --- | --- | --- |
|  |  | **Excellent** | **Satisfactory** | **Average** | **Poor** | **(*p*-value)** | **Sleep curtailment (< 8h)** | **Adequate sleep**  **(≥ 8h)** | **(*p*-value)** | **<30 min** | **≥30 min** | **(*p*-value)** |
| Gender | Boys | 271 | 156 | 59 | 29 | 7.944  (0.047*) | 348 | 167 | 13.21  **(0.001**)** | 476 | 39 | 0.495  (0.482) |
|  | Girls | 208 | 157 | 75 | 27 |  | 373 | 94 |  | 437 | 30 |  |
| Age | 13-15 | 220 | 104 | 24 | 9 | 49.959  (0.000***) | 194 | 163 | 104.64  **(0.000***)** | 333 | 24 | 0.079  (0.778) |
|  | 16-19 | 259 | 209 | 110 | 47 |  | 527 | 98 |  | 580 | 45 |  |
| Family residence | Rural | 195 | 85 | 35 | 16 | 20.603  (0.000***) | 232 | 99 | 2.839  (0.092) | 306 | 25 | 0.212  (0.645) |
|  | Urban | 284 | 228 | 99 | 40 |  | 489 | 162 |  | 607 | 44 |  |
| Paternal education level | Junior high school or below | 169 | 112 | 43 | 21 | 10.827  (0.094) | 252 | 93 | 2.901  (0.408) | 312 | 33 | 6.681  (0.083) |
|  | High school or secondary technical school | 169 | 116 | 49 | 18 |  | 261 | 91 |  | 335 | 17 |  |
|  | Junior college | 96 | 43 | 23 | 8 |  | 118 | 52 |  | 157 | 13 |  |
|  | Bachelor or above | 45 | 42 | 19 | 9 |  | 90 | 25 |  | 109 | 6 |  |
| Maternal education level | Junior high school or below | 190 | 117 | 52 | 22 | 13.594  (0.138) | 282 | 99 | 6.222  (0.101) | 352 | 29 | 5.703  (0.68) |
|  | High school or secondary technical school | 151 | 75 | 38 | 16 |  | 203 | 77 |  | 265 | 15 |  |
|  | Junior college | 96 | 88 | 30 | 9 |  | 155 | 68 |  | 204 | 19 |  |
|  | Bachelor or above | 42 | 33 | 14 | 9 |  | 81 | 17 |  | 92 | 6 |  |
| Family economic status | Low | 91 | 54 | 26 | 15 | 3.161  (0.788) | 136 | 50 | 0.478  (0.787) | 173 | 13 | 0.05  (0.975) |
|  | Middle | 358 | 239 | 98 | 38 |  | 541 | 192 |  | 681 | 52 |  |
|  | Good | 30 | 20 | 10 | 3 |  | 44 | 19 |  | 59 | 4 |  |
| BMI | Normal | 383 | 262 | 86 | 38 | 34.751  (0.000***) | 569 | 200 | 0.592  (0.744) | 739 | 30 | 55.105  **(0.000***)** |
|  | Overweight | 71 | 37 | 34 | 8 |  | 107 | 43 |  | 125 | 25 |  |
|  | Obesity | 25 | 14 | 14 | 10 |  | 45 | 18 |  | 49 | 14 |  |

Note: *, p<0.05; **, p<0.01; ***, p<0.001.

**Supplementary table 3. Associations between sociodemographic variables and**

**dietary disorder level**

| **Variables** | **Group** | **Dietary disorder level** | | | **χ²** |
| --- | --- | --- | --- | --- | --- |
|  |  | **Low** | **Moderate** | **High** | **（*p*-value）** |
| Gender | Boys | 159 | 206 | 150 | 1.885（0.39） |
|  | Girls | 127 | 190 | 150 |  |
| Age | 13-15 | 134 | 146 | 77 | 28.48（**0.000*****） |
|  | 16-19 | 152 | 250 | 223 |  |
| Family residence | Rural | 119 | 125 | 87 | 11.778（**0.003****） |
|  | Urban | 167 | 271 | 213 |  |
| Paternal education level | Junior high school or below | 100 | 136 | 109 | 6.233  （0.398） |
|  | High school or technical secondary school | 99 | 149 | 104 |  |
|  | Junior college or above | 60 | 64 | 46 |  |
|  | Bachelor or above | 27 | 47 | 41 |  |
| Maternal education level | Junior high school or below | 112 | 147 | 122 | 21.746（**0.001****） |
|  | High school or technical secondary school | 93 | 100 | 87 |  |
|  | Junior college or above | 54 | 117 | 52 |  |
|  | Bachelor or above | 27 | 32 | 39 |  |
| Family economic status | Low | 52 | 78 | 56 | 0.894（0.925） |
|  | Moderate | 216 | 295 | 222 |  |
|  | High | 18 | 23 | 22 |  |
| BMI | Normal | 218 | 299 | 252 | 12.975（**0.011***） |
|  | Overweight | 54 | 67 | 29 |  |
|  | Obesity | 14 | 30 | 19 |  |

Note: *, p<0.05; **, p<0.01; ***, p<0.001.
